# Updating Nursing Competencies in Primary Healthcare in Albania; Transforming Roles Through Tailored Education

**DOI:** 10.3389/ijph.2021.1604085

**Published:** 2021-09-17

**Authors:** Jonila Gabrani

**Affiliations:** ^1^ Faculty of Medicine, University of Basel, Basel, Switzerland; ^2^ Swiss Centre for International Health, Swiss Tropical and Public Health Institute (Swiss TPH), Basel, Switzerland

**Keywords:** education, nurses, PHC, competencies, roles

**The IJPH series “Young Researcher Editorial” is a training project of the Swiss School of Public Health.**

Patients are more satisfied and health care costs are lower if primary health care [PHC] nurses are competent. Increases in both the quality of public and private PHC service and public demand [1, 2] have sparked discussions about nurses’ role in PCH. This essay argues that delivering holistic, patient-centred, integrated PHC services requires redefining the roles of nurses and strengthening their clinical and attitudinal competencies, including training in the social dimensions of care.

Countries such as the United States, Australia, and Canada have quickly updated their nursing profiles and introduced new competencies for nurses working in PHC. In the United States and Australia, PHC nurses are the first point of contact for patients and help prevent and manage chronic conditions [1]. In Canada, nurses autonomously diagnose, order, and interpret diagnostic tests and prescribe pharmaceuticals [3]. In other parts of the world, like the Western Balkan region, the transition to new nursing profiles has been slower [4], but international agencies are offering support to nations that want to transform nursing health education to improve communication, teamwork, critical thinking, digital [5, 6], and social skills among PHC nurses [1], and to establish interprofessional PHC teams.

In Albania, a Western Balkan country, the post-communist system still relies heavily upon the ultimate authority of doctors. However, Albania needs competent PHC nurses who combine professional skills, knowledge, and values with clinical competencies. Nurses of this caliber are necessary to [i] combat the steady rise of non-communicable diseases [NCDs], [ii] address the needs of an aging population, and [iii] compensate for the shortage of doctors. In Serbia, there is still no nurse specialization in PHC and their role is limited to registering patients and assisting doctors with paperwork. Most are not trained to provide any counseling services for patients with NCDs [7].

Even though the necessary competencies must be taught in educational institutions and through professional training, nursing education in Western Balkan colleges, universities, workplaces, and health systems has not yet been upgraded to meet the educational standards set by the European Union [EU]’s directive on regulated professions [4]. For example, in Albania, in the early 2000s, the ‘Bologna system’ was introduced at the university level, and students can now earn a Bachelor’s degree in three academic years. New professional specializations emerged, and Bachelor’s programs now graduate speech therapists, physical therapists, and laboratory technicians, among others. Yet diplomas in ‘general nursing’ remain the most preferred.

Accredited public and private universities now offer a variety of curricula in nursing at the Bachelor’s level, though the available modules may differ substantially between public and private universities [8]. While the complexities of modern healthcare do create the need for diverse training programs for nurses and a variety of competency profiles, the lack of standardization at the Bachelor’s level makes a transfer to other universities difficult, impeding students’ mobility.

Basic nursing education in both the public and the private systems should be standardized in Albania and the rest of the region, meaning training and the educational process should be upgraded [4]. At the Master’s level, professional degrees should be tailored around real-world healthcare settings and the burden of disease. In 2020, the Faculty of Technical Medical Sciences collaborated with the “Health for All” project in Albania. “Health for All” supported their efforts to provide practice-focused training to nursing students in a new professional Master’s program called Family Health Nurse. The effort was also funded by the Swiss Agency for Development and Cooperation and implemented through the Swiss Tropical and Public Health Institute. This initiative aligned with Albania’s national health agenda [PHC strategy 2020–2025], and the population’s growing need for high-quality public and private PHC services [9, 10]_._

However, developing competencies and skills is not enough. The roles and positions of nurses on the PHC team also need to be redefined. This redefinition may change PHC delivery models and raise the status of nurses in the health system. But new nursing models challenge current practice in Albania and the region, where nurses’ roles remain traditional [4]. In Albania, some may resist the idea that nurses should take over tasks related to managing NCDs. Nurses might find it challenging to take on these new tasks until they receive more training and support from physicians.

Despite these potential obstacles, the benefits of tailoring and standardizing nursing education are clear. Better utilizing and integrating nurses in the PHC system is a prerequisite for meeting current and future challenges to the health system. In addition to updating the current curricula, creating interesting job facets for nurses, and offering improved continuing education and training courses, we must ensure that the PHC system fosters teamwork and respectful relationships between all members.
